# Data-Driven Virtual Screening of Conformational Ensembles of Transition-Metal Complexes

**DOI:** 10.1021/acs.jctc.5c00303

**Published:** 2025-05-09

**Authors:** Sára Finta, Adarsh V. Kalikadien, Evgeny A. Pidko

**Affiliations:** Inorganic Systems Engineering, Department of Chemical Engineering, Faculty of Applied Sciences, 2860 Delft University of Technology, Van der Maasweg 9, 2629 HZ Delft, The Netherlands

## Abstract

Transition-metal complexes serve as highly enantioselective homogeneous catalysts for various transformations, making them valuable in the pharmaceutical industry. Data-driven prediction models can accelerate high-throughput catalyst design but require computer-readable representations that account for conformational flexibility. This is typically achieved through high-level conformer searches, followed by DFT optimization of the transition-metal complexes. However, conformer selection remains reliant on human assumptions, with no cost-efficient and generalizable workflow available. To address this, we introduce an automated approach to correlate CREST­(GFN2-xTB//GFN-FF)-generated conformer ensembles with their DFT-optimized counterparts for systematic conformer selection. We analyzed 24 precatalyst structures, performing CREST conformer searches, followed by full DFT optimization. Three filtering methods were evaluated: (i) geometric ligand descriptors, (ii) PCA-based selection, and (iii) DBSCAN clustering using RMSD and energy. The proposed methods were validated on Rh-based catalysts featuring bisphosphine ligands, which are widely employed in hydrogenation reactions. To assess general applicability, both the precatalyst and its corresponding acrylate-bound complex were analyzed. Our results confirm that CREST overestimates ligand flexibility, and energy-based filtering is ineffective. PCA-based selection failed to distinguish conformers by DFT energy, while RMSD-based filtering improved selection but lacked tunability. DBSCAN clustering provided the most effective approach, eliminating redundancies while preserving key configurations. This method remained robust across data sets and is computationally efficient without requiring molecular descriptor calculations. These findings highlight the limitations of energy-based filtering and the advantages of structure-based approaches for conformer selection. While DBSCAN clustering is a practical solution, its parameters remain system-dependent. For high-accuracy applications, refined energy calculations may be necessary; however, DBSCAN-based clustering offers a computationally accessible strategy for rapid catalyst representations involving conformational flexibility.

## Graphical abstract

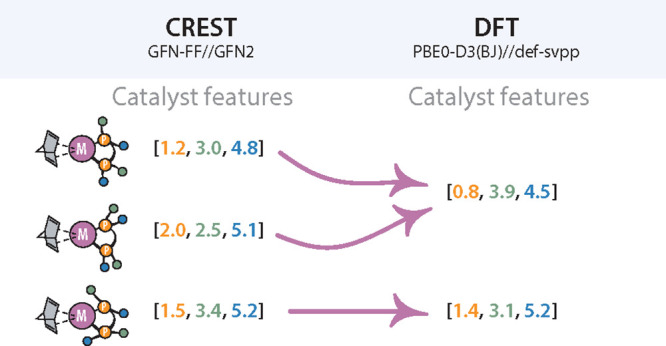

## 1 Introduction

Data-driven approaches are reshaping many domains of chemical research, offering unprecedented opportunities for deeper analysis and accelerating chemical discoveries.^1–4^ In particular, data-driven models hold great promise in developing predictive strategies for rational catalyst design. These approaches can facilitate and accelerate the implementation of greener, selective, and scalable sustainable chemical transformations using tailor-made homogeneous catalysts for the fine chemical and pharmaceutical industries.^5–8^ The vast chemical space established by the transition metal complexes with their versatile and highly tunable ligands has been navigated by the homogeneous catalysis community over the last century in the search for precise control over catalytic chemistry and catalyst behavior.^9,10^ The extensive diversity of the ligands and their diverging behavior depending on the conditions and the metal type present a formidable combinatorial challenge, complicating the rational exploration of the transition-metal (TM) chemical space in the search for the optimal catalyst. The traditional largely heuristic and serendipity-driven catalyst development methods are increasingly complemented by high-throughput techniques, which generate systematic broader data sets, thus expanding the scope and capabilities of the analysis.^9,11–13^ Such data sets can be analyzed using advanced computational methodologies including QSAR/QSPR, machine learning, and statistical tools, to facilitate the identification of correlations between the molecular characteristics and the catalytic behavior, accelerating and guiding the search for the optimal catalyst.^14–17^

One of the major challenges in applying data-driven algorithms to catalysis lies in creating accurate, computer-readable representations of molecules.^18–20^ The universality of the resulting models and their predictive capabilities heavily depend on how well the specific features included in the molecular representation capture the fundamental characteristics and behavior of the catalytic species that ultimately determine the reactivity.^14–17,19,20^ Although most models focus on features associated with static molecular representations, incorporating properties calculated on a conformer ensemble into the featurization step has gained traction as a means to better capture the fluxionality of molecular systems under reaction conditions and improve predictive accuracy.^21,22^ Both experimental and computational studies underscore the importance of catalyst conformations for catalytic activity and enantioselectivity,^23–25^ as different conformers may exhibit unique steric effects and energy profiles.^21,26^ Given the sensitivity of physical-chemical properties to structural variations, including conformational effects is essential for accurate feature acquisition.^7,27,28^

However, identifying suitable conformer searching algorithms for TM complexes remains challenging due to the complexity of these systems.^29,30^ TM complexes are large and feature a wide variety of bond types, and there is a lack of fast, efficient methods that can effectively handle such systems, particularly in the context of high-throughput exploration of highly fluxional chemical environments. Broadly exploring possible conformations for large complexes comes with significant computational costs.^31,32^ On the other hand, relying on chemical intuition to select conformers can introduce human bias, often leading to inaccurate representations and neglect of critical conformational effects.^31,33^

To achieve descriptors of conformers that accurately capture their physical-chemical properties, DFT-level calculations are typically utilized.^34^ Quantum chemistry-based conformer searching methods, such as AARON,^35^ use DFT calculations to produce precise conformer results, though they come at a high computational cost. As a result, many workflows begin with a less costly conformer exploration using force field or semiempirical methods.^22,36^ Common force field-based algorithms include RDKit,^37^ OpenBabel,^38^ and MOLASSEMBLER,^39^ while CREST (Conformer–Rotamer Sampling Tool) is a widely used tool applying GFNn-xTB tight-binding semiempirical methods.^40^ Examples of methods for nonbiased exploration of stereochemistry that utilize RDKit or Openbabel in the back-end are Architector^41^ and MACE.^42^ In most workflows, the ensembles generated in these initial steps are then refined with DFT to enhance accuracy.^43,44^

Selecting which conformers to refine is not straightforward. Ideally, the goal is to identify conformers that correspond to local minima on the DFT potential energy surface. A logical approach might involve selecting conformers with low relative energy within the ensemble based on energies calculated by a semiempirical method. However, a significant challenge with current conformer searching methods is the unreliable energy ranking within the ensembles. Previous studies have highlighted the limitations of classical force fields (FF) and semiempirical methods in accurately predicting energy ordering and global minima compared to DFT-level calculations.^11,29,30,45^ Consequently, relying solely on energy values for filtering could risk excluding important low-energy conformers that would otherwise be identified on the DFT potential energy surface. In CREST, an alternative option is based on principal component analysis (PCA) clustering, which performs PCA and then clusters conformers based on dihedral angles. However, as reported in the CREST documentation, the algorithm cannot accommodate noncovalent bonds, which often occur in transition metal complexes. Furthermore, the algorithm applies *k*-means clustering, where the number of clusters is a predetermined variable. Another commonly used filtering approach is the CENSO workflow.^34,46^ This screening approach uses the obtained CREST ensemble as input and performs prefiltering based on the energies obtained from DFT single-point calculations. The remaining conformers undergo DFT geometry optimization, during which several filtering steps are included based on energy thresholds. The final ensemble is obtained through a pruning step based on the Gibbs free energy. Although effective, this approach is based on constant re-evaluation of the energy of each conformer, increasing the computational cost with increasing flexibility of the molecule.

In our work, we aimed to investigate a practical and generic approach for streamlining the generation of a DFT-based conformer ensemble from lower-level ensembles in the context of high-throughput computational catalyst screening. Hence, we sought to answer the following question: what filtering method or combination of methods allows for a conformer selection workflow in which computational cost is kept low while a high accuracy is maintained? Several high-throughput and automated filtering approaches for conformer ensembles were investigated. Conformer ensembles were generated for 24 Rh-based catalysts originating from our previous work, utilizing bisphosphine ligands.^47^ Parameters from the CREST-based ensemble were used to filter and the DFT refined ensemble was used as a ground truth, which enabled the quantification of the effectiveness of a filtering method. More specifically, by establishing a set of molecular descriptors we aimed to enable a data-driven filtering approach. Two data-driven filtering approaches were tested, the first one was a principal component analysis based on a set of steric, geometric and electronic descriptors calculated on the conformer ensemble. The second data-driven filtering approach was a heuristic approach based on the relative values of selected geometric and steric descriptors. Finally, a density-based clustering of relative energy and root-mean square deviation (RMSD) values was performed, constituting the simplest filtering approach investigated in this work.

## 2 Computational Methods

### 2.1 Conformer Generation and Filtering Workflow

This study is based on a data set from our previous research on Rh-based catalyst employing primarily bidentate ligands.^47^ From that study, 24 catalyst structures were randomly selected as the starting point for the current research. Each structure featured a Rh metal center with a bisphosphine ligand attached to it. A norbornadiene (NBD) moiety was coordinated trans to the bisphosphine ligand to reflect the precatalyst state.^47^ These structures are referred to as L1 to L24, where the number corresponds to the ligand identity. Visualizations of the ligands are available in the Data Availability section. In this study, the digital representation of the catalyst structures, i.e., in XYZ and MDL Molfile format, was utilized for further investigation via conformer searching and filtering methods.

An overview of the workflow for this study is presented in Figure 1, in which two stages can be identified: the first stage involves the generation of CREST conformer sets and the subsequent optimization based on DFT. This data set served as a platform to test our conformer ensemble filtering approaches. The second stage explores various methods aimed at accurately modeling the contents of the refined DFT-based ensembles using features and parameters derived from the CREST ensembles.

**Figure 1:**
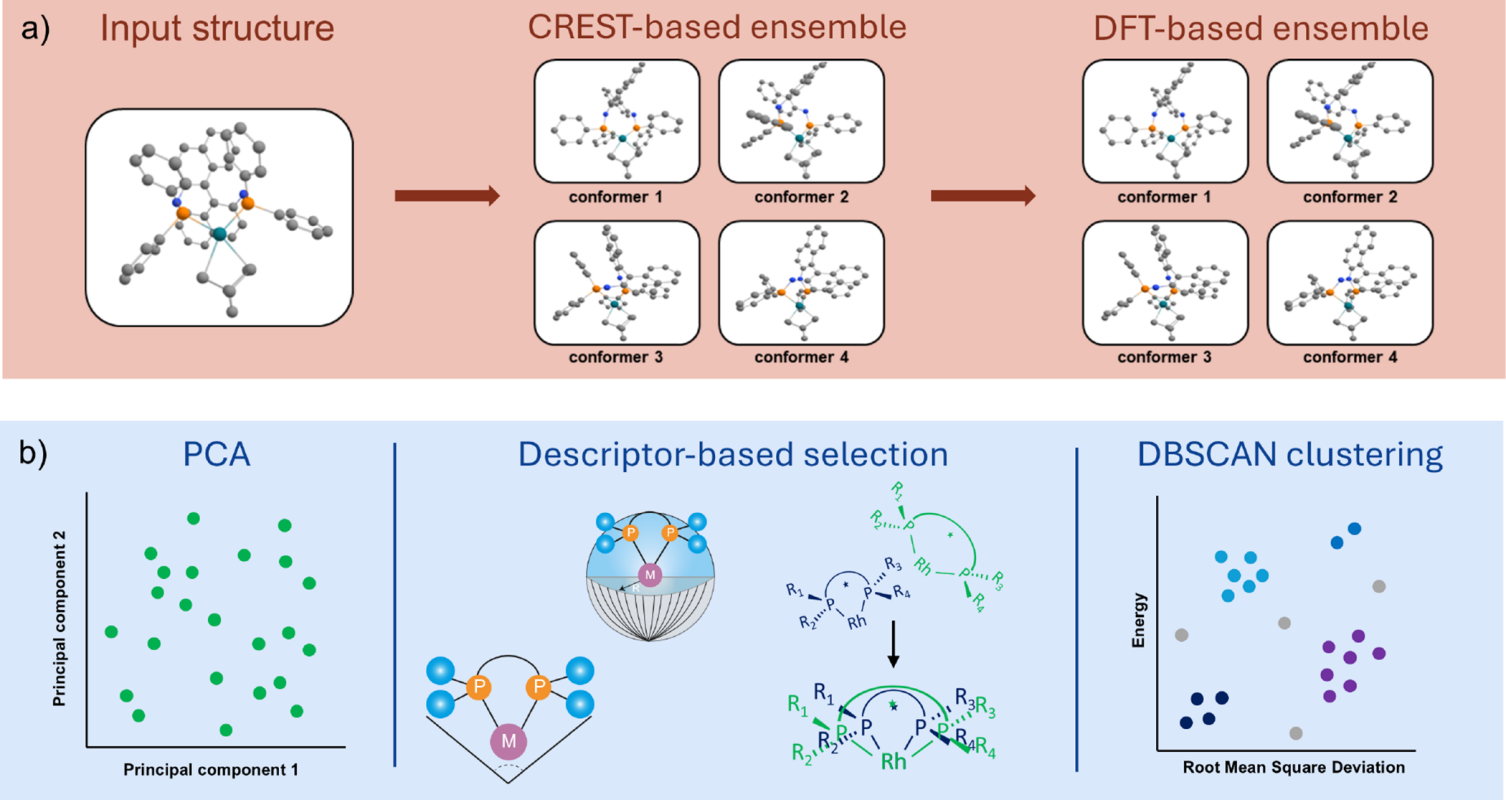
Overview of the applied workflow with a representative illustration of the Rh-based catalyst structures. (a) Creation of conformer ensembles via CREST and subsequent DFT refinement. (b) Various methods were tested to relate a representation of the CREST-based conformer ensemble to the DFT-based refined ensemble.

### 2.2 Quantum Chemical Methods

For stage one of the workflow, conformer generation and exploration were conducted using the Conformer-Rotamer Ensemble Sampling Tool (CREST) version 2.12 ^40,48^ and xTB version 6.4.0.^49^ CREST calculations were performed on all 24 Rh-based structures using Cartesian coordinates (*.xyz file) as input geometries for conformer ensemble creation. The GFN2-xTB//GFN-FF hybrid potential was chosen for its accurate performance at reasonable computational costs and universal applicability.^11^ For readability purposes, the CREST­(GFN2-xTB//GFN-FF)-generated conformer ensembles are referred to as ‘CREST-based conformer ensembles’. Conformers generated by CREST were subsequently preprocessed using the MORFEUS Python package (version 0.7.1). The python package readily takes obtained CREST output folders as an input, which accommodates further filtering and analysis. To enable this, an explicitly added connectivity matrix was extracted from an MDL Molfile. Afterward, structures that exhibited changes in chirality relative to the original input structure were removed from the ensemble.

The resulting CREST-based conformer ensembles were refined via DFT geometry optimization, performed using Gaussian 16 C.02.^50^ The PBE0-D3­(BJ)/def2-SVPP level of theory^51–53^ was applied, known for its reliable accuracy and efficiency for the description of TM complexes.^11,54,55^ The nature of each stationary point was confirmed via frequency analysis. Thermochemical parameters (e.g., ZPE, finite temperature corrections and entropy contributions to Gibbs free energies) were computed from analytical frequencies (Hessian) at 298.15 K and 1 atm. For conformers displaying imaginary frequencies, the pyQRC Python script (version 1.0.3)^56,57^ was employed to generate revised input geometries, which were then reoptimized with the same DFT settings. Conformers that retained imaginary frequencies after two attempts at reoptimization were excluded from further evaluation.

### 2.3 Data Analysis

The core objective of this study is to identify a subset of conformers from the CREST ensemble that best represent the DFT ensemble, using DFT-derived energy values from stage I (Figure 1a) as the reference. The main part of the workflow (stage II, Figure 1b) involves the evaluation of various algorithms selection methods to determine their effectiveness in capturing the most relevant conformers. In this context, assuming chemical accuracy of ca. 5 kJ/mol, conformers within this energy range were considered indistinguishable in the DFT ensemble.^58^ An automated script was developed to perform this task, followed by additional manual adjustments. The finalized DFT ensembles are available in the Data Availability section.

Molecular descriptors of the CREST-based conformers were calculated using the OBeLiX (Open Bidentate Ligand eXplorer) open-source computational package.^7^ With the MORFEUS conformer ensemble object as input, a total of 37 descriptor values for each individual conformer including steric, geometric and electronic properties were calculated. A comprehensive list of these descriptors is provided in the Data Availability section. Additionally, structural differences between conformers were incorporated into the analysis using the heavy-atom root-mean-square deviation (RMSD) relative to the first (lowest CREST energy) conformer in the ensemble. The RMSD calculations were performed with the MORFEUS package using its default settings.

As shown in Figure 1b, three approaches were used to identify a subset of conformers from the CREST ensembles that accurately represent the DFT ensemble: a principal component analysis (PCA), a molecular descriptor-based selection and a DBSCAN clustering of relative energy and RMSD values. For the PCA, the data set of selected molecular descriptors supplemented by the RMSD values of the conformers was utilized. To standardize the data set, a standard scaling procedure was applied to the descriptors, ensuring uniform data ranges with a mean of zero and a standard deviation of one. This analysis focused on the first two principal components only. In the second approach, molecular descriptor-based selection methods, certain steric and geometric properties, such as the cone angle and buried volume, were used for conformer selection. This approach ensures that the selected conformer set includes conformers with varied steric and geometric profiles, including the extremes that define distinct accessible value ranges for these properties.^59^ Based on this, it was chosen to select CREST-based conformers with the minimum and maximum values for both buried volume (calculated at the metal center with radius 4 Å) and cone angle. The third approach applied DBSCAN clustering on the relative energy and RMSD values of conformers within the ensemble, with the minimum cluster size parameter set to 2, while the distance-to-centroid parameter was further optimized based on model performance.

The investigated methods were primarily evaluated by a confusion matrix. The following approach was used to determine the parameters of the confusion matrix:

- True negative (TN): The number of conformers that are correctly eliminated by the algorithm: their DFT minima are already represented by other conformers in the predicted subset, making them redundant to cover the DFT ensemble.
- False negative (FN): The number of conformers that are incorrectly eliminated by the algorithm: their DFT minima are not represented by other conformers in the predicted subset, making them necessary to cover the DFT ensemble.
- False positive (FP): The number of conformers that are incorrectly included in the predicted subset by the algorithm: their DFT minima are already represented by other conformers, making them redundant to cover the DFT ensemble.
- True positive (TP): The number of conformers that are correctly included in the predicted subset by the algorithm: their DFT minima are not represented by other conformers, making them necessary to cover the DFT ensemble.

The chosen evaluation parameters were true negative (TN) and false negative (FN) values. In a well-performing model, TN is maximized to ensure that all redundant conformers are removed, while FN is minimized to ensure that no DFT minimum is overlooked.

### 2.4 Validation

A data set from our previous study, in which both CREST-based conformer ensembles and their DFT-optimized structures were available, was used for further validation purposes. This data set also consisted of Rh-based catalysts with bisphosphine ligands, but instead of using the precatalyst form with NBD coordinated to the metal center, a methyl 2-acetamidoacrylate substrate was coordinated to Rh. Based on the ligand-substrate configurations, four different coordination modes are possible of which two are more sterically restricted, and two are less sterically restricted.^11^ Our workflow was tested on the 44 CREST ensembles from 11 different ligands. Generally, the substrate coordination gives the structures more flexibility compared the precatalyst form with NBD. This makes the conformer ensembles extend beyond the possibilities of manual analysis within the restrictions of reasonable labor costs and thus serves as a representative case study where high-throughput conformer analysis would be useful.

## 3 Results and Discussion

### 3.1 Conformer Search and DFT Geometry Optimization

The refinement of all conformers at a high level of theory after low-level conformational searches can significantly increase computational cost without justified gains. This can be demonstrated by comparing the conformer ensembles generated by CREST and refined at the DFT level of theory. CREST, employing xTB, generally predicts much greater conformational freedom, characterized by a broader range and higher number of individual conformers than those retained after DFT optimization (Figure 2). Specifically, while CREST generated a total of 678 conformers across the 24 input structures, the DFT ensembles retained a considerably smaller subset of these conformers. Among the 24 ensembles analyzed, the average number of conformers per ensemble at the xTB level was 23, which was reduced to an average of only 2 conformers per ensemble after DFT refinement. The CREST ensembles exhibited considerable variation in the number of conformers obtained; for example, the ensembles for L7 and L23 comprised only eight conformers, while the largest ensemble, L18, contained 78 conformers. Following DFT refinement, both L23 and L18 yielded a single conformer in the DFT ensemble, whereas the ensemble for L7 contained two conformers. The large reduction observed in ensemble size after DFT refinement is in line with our previous observations,^11^ which indicate that the size of conformer ensembles decreases greatly after the DFT refinement.

**Figure 2:**
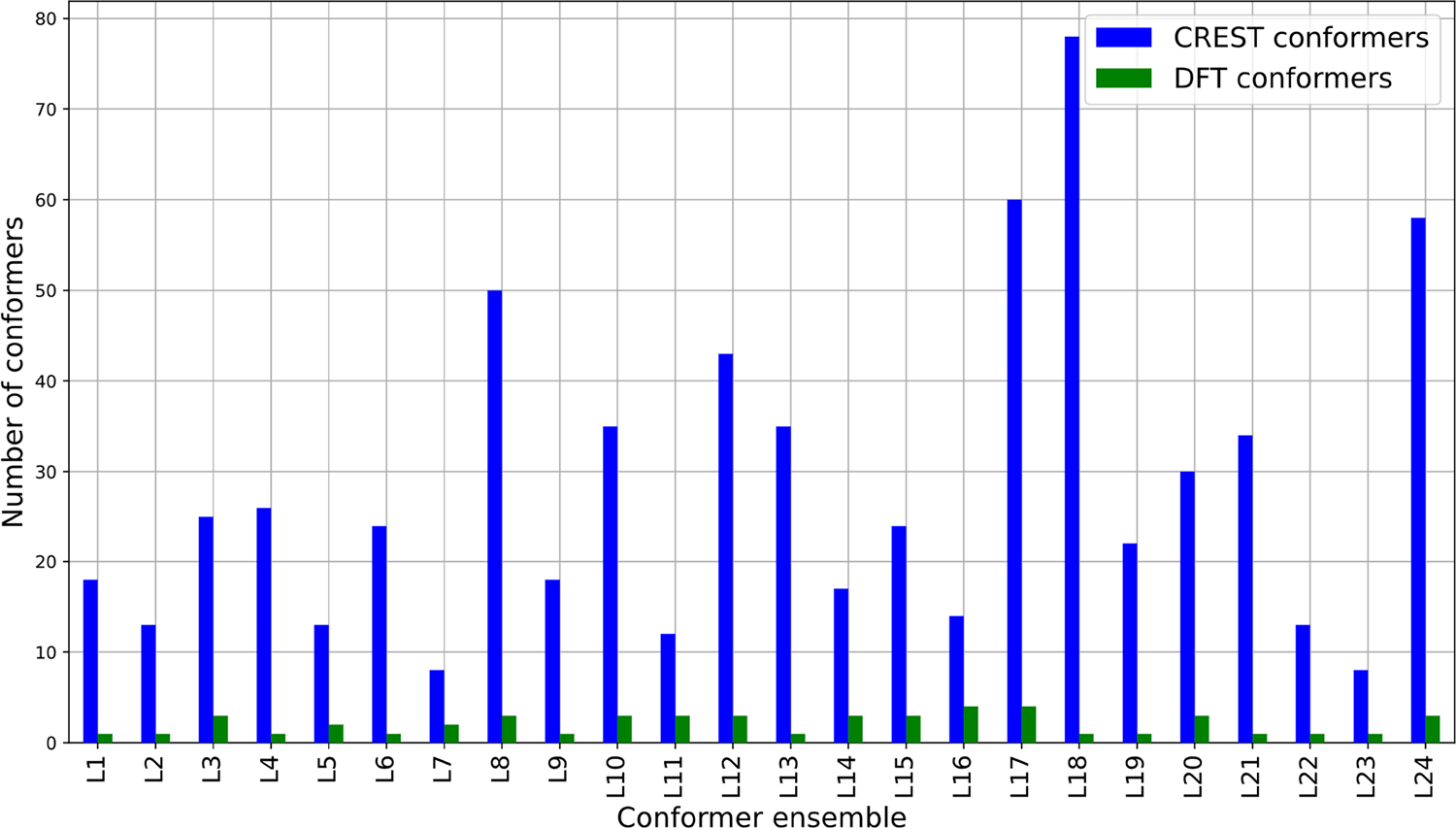
Comparison of the number of conformers obtained from both CREST and DFT calculations. The number of conformers in each ensemble is indicated in blue for the CREST ensembles and in green for the DFT ensembles. The ensembles were named according to ligand numbering, which can be found in the list of ligands in the Data Availability section.

To investigate this in more detail, four representative ensembles are examined: L3, L8, L17, and L24. Figure 3 compares the relative stabilities of the conformers from CREST at the xTB level (Δ*E*_xTB_) and after DFT refinement (Δ*E*_DFT_). The broad conformer space predicted by CREST collapses to only a few distinct conformers after DFT optimization (Figure 3). Furthermore, the relative stabilities predicted at the xTB level do not correlate with those computed at the DFT level. For example, in ensemble L17, the conformer ranked as lowest-energy by CREST is 21 kJ/mol higher than the lowest DFT energy conformer. Similarly, in ensemble L8, the CREST lowest-energy conformer has a higher energy by 19 kJ/mol compared to the lowest DFT conformer.

**Figure 3:**
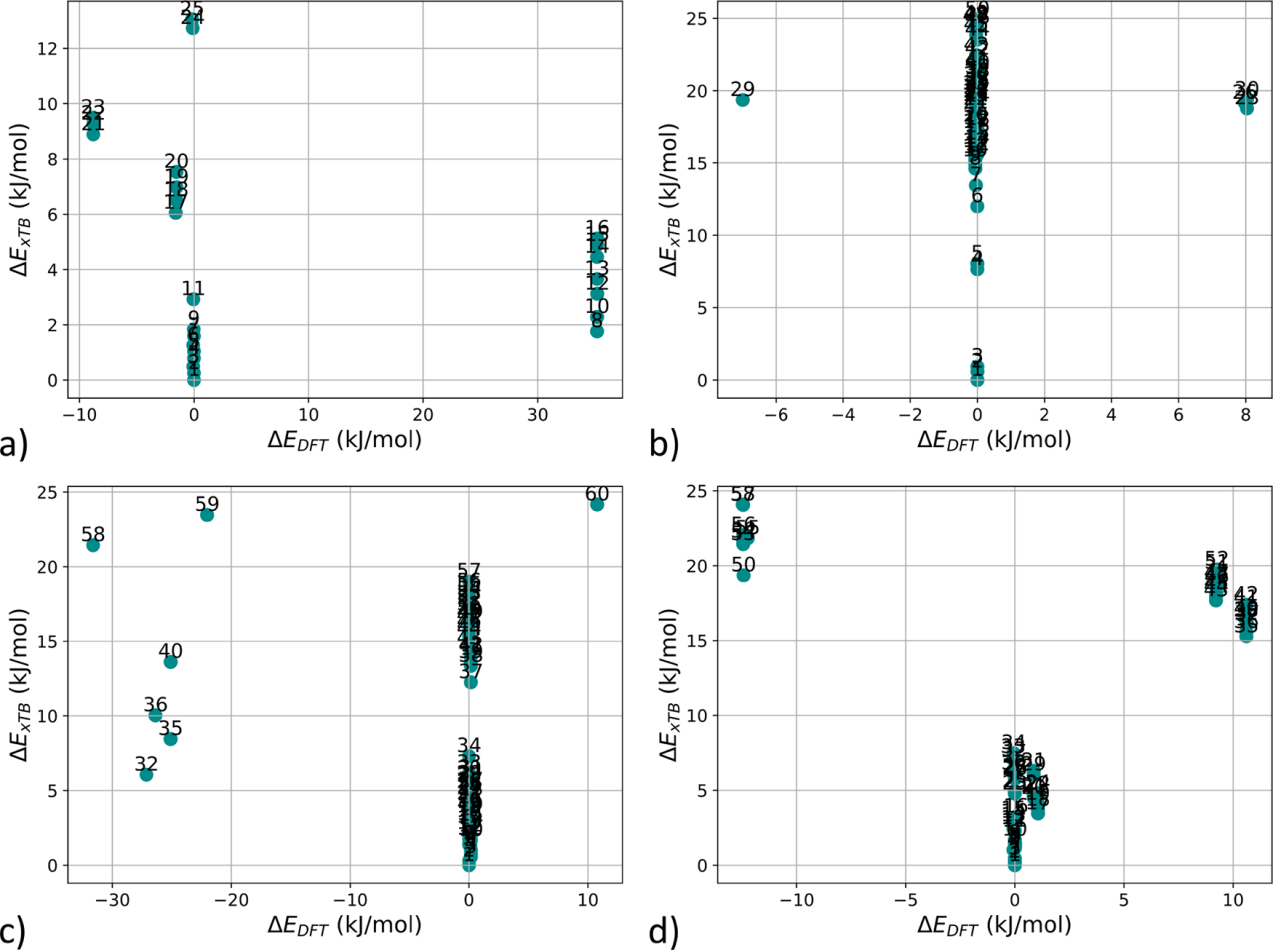
DFT and xTB energies relative to the conformer with the lowest xTB energy of ensemble L3 (a), ensemble L8 (b), ensemble L17 (c), and ensemble L24 (d).

These examples highlight a key point: the apparent differences in flexibility predicted by the two methods stem from the fact that many of the CREST conformers, even those with large energy differences, converge to the same DFT conformer after optimization. This comparison reveals that the flexibility of the complexes obtained by xTB is overestimated, resulting in a much smaller conformer space at the higher level of theory.

The discrepancy between the conformer spaces predicted by xTB and DFT highlights a significant challenge when lower-level methods are utilized for conformer selection prior to further refinement: if one selects only the global minimum or a limited number of low-lying CREST conformers for subsequent refinement and physical-chemical descriptor calculation, there is a high probability of misrepresenting the actual higher-level ensemble. In the absence of more sophisticated conformer selection strategies, this approach risks overlooking relevant structural diversity and introducing bias into the results as highlighted by Laplaza et al.^33^ Consequently, computational resources may be wasted, and a comprehensive understanding of the system’s true conformational space may not be achieved.

### 3.2 Methods Based on Descriptors

We introduce a systematic analysis framework to establish a more robust connection between the xTB and DFT conformer ensembles, with the objective of automating conformer selection while ensuring the retention of all unique configurations. To evaluate the correlation between the CREST-based ensemble and its DFT-optimized counterpart, we calculated a set of descriptors on the CREST conformer ensemble. These descriptors, including relative energy, RMSD, cone angle, and buried volume, were used to assess the effectiveness of filtering methods in generating a subset of conformers that closely mirror the DFT ensemble. The RMSD and Δ*E*_xTB_ values of the conformers were employed to eliminate redundant conformers through geometry and energy pruning methods as implemented in the MORFEUS Python package. Similar approaches are implemented in the AQME package.^60^ The RMSD pruning method targets structural redundancy, based on the hypothesis that conformers with similar geometries, indicated by an RMSD within 0.35 Å of the lowest-energy conformer, are likely to converge to the same DFT minimum upon refinement. In contrast, the energy pruning method eliminates conformers with relative xTB-based energies exceeding a threshold of 12.55 kJ/mol (3.0 kcal/mol), suggesting that conformers with close relative energies may exhibit similar stabilities and thus contribute similarly to the conformational space. To further refine the conformer selection, we also considered geometric descriptors such as cone angle and buried volume, which are widely used to characterize the steric and geometric properties of catalysts. These parameters were selected based on the hypothesis that they would capture conformational variability in steric profiles that is not necessarily reflected in electronic properties.^59,61^ The cone angle and buried volume are particularly sensitive to steric variations, which are crucial for understanding structural differences in catalytic environments. Therefore, we hypothesized that CREST-based conformers with extreme cone angles and buried volumes are more likely to converge to distinct DFT minima, reflecting significant conformational differences.

To validate the use of cone angle and buried volume as key descriptors for distinguishing unique DFT minima, an initial analysis was conducted across the 24 conformer ensembles. Out of the 24 ensembles analyzed, 13 showed more than one DFT minimum. In 11 of these cases, the conformers with the highest and lowest buried volumes converged to distinct DFT minima, while in 2 cases (ensembles L3 and L17), they converged to the same minimum. For the cone angle descriptor, the conformers with the highest and lowest values converged to the same DFT minimum in 3 instances (ensembles L3, L8, and L12). This suggests that the combination of these two descriptors successfully differentiated at least two DFT minima in 12 of the 13 cases, providing a basis to utilize them in descriptor-based filtering methods. Three different pruning methods were used prior to the selection process, as shown in Figure 4. These methods vary by pruning approach: RMSD pruning (method 1), energy pruning (method 2), and a combined approach using both RMSD and energy pruning (method 3). In each method, the selected conformers retained were those with the highest and lowest cone angle and buried volume within the CREST ensemble.

**Figure 4:**
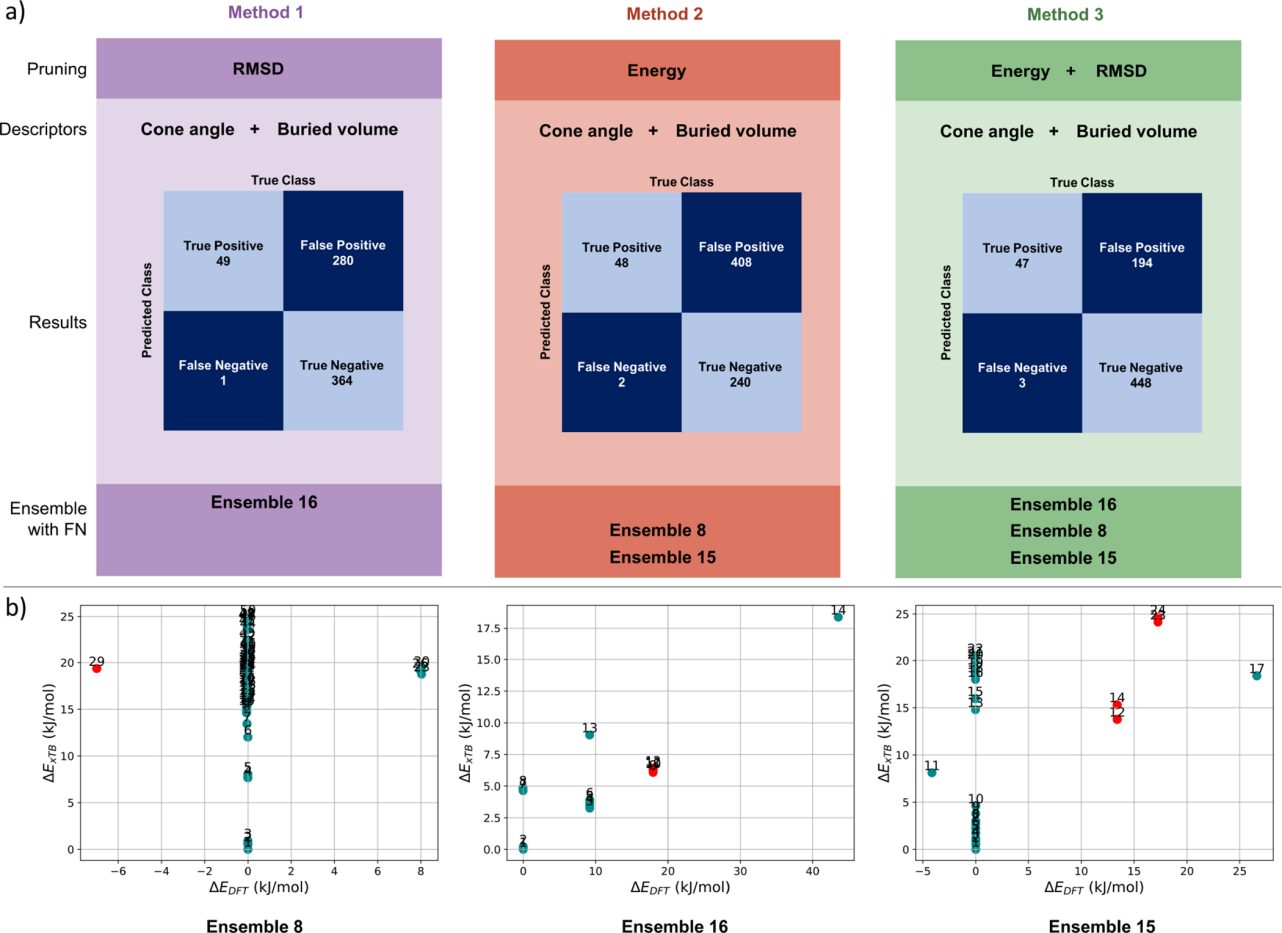
(a) Scheme and results of three descriptor-based filtering approaches. In method 1 (left), RMSD pruning is applied; in method 2 (center), energy pruning is applied; and in method 3 (right), both RMSD and energy pruning are combined. Confusion matrices for each method are shown, highlighting the primary assessment parameters: false negatives (FN) and true negatives (TN). Additionally, each ensemble where a DFT minimum is missed (FN) is indicated. (b) CREST-DFT relative energy plots are provided for three ensembles, where DFT minima are potentially missed, with conformers associated with a missed DFT minimum marked in red.

In an ideal case, as many redundant conformers as possible are eliminated (true negatives) while minimizing the number of unique DFT minima missed (false negatives). Since the same descriptors were applied in for all selection methods, but the pruning methods differed, evaluating these parameters highlights the relative effectiveness of each pruning approach in balancing computational efficiency with accuracy.

Across the 24 CREST ensembles analyzed, a total of 644 redundant and 50 significant conformers were identified. The RMSD pruning method removed 364 (56%) of the redundant conformers, while the energy pruning eliminated only 240 (37%). A notable distinction between the two approaches is that RMSD pruning missed only one DFT minimum (in ensemble L16), while energy pruning failed to capture two DFT minima (one each in ensembles L8 and L15). Figure 4 shows that for ensembles L15 and L16, the missed DFT minima are not the lowest energy conformers, whereas in ensemble L8, the global DFT minimum is missed. Therefore, applying RMSD pruning is more effective in both reducing redundant conformers and capturing all minima of the DFT ensemble. This indicates that conformers that show strong structural similarities in the CREST space are more likely to converge into the minimum upon further geometry refinement than conformers that show similar energy values. In method 3, which combines both RMSD and energy pruning, all three of the previously mentioned DFT minima were missed (one each from ensembles L8, L15, and L16). However, this combined approach successfully removed 448 redundant conformers, representing 70% of the total redundancies. This indicates that the combined pruning method offers an effective option for applications where maximizing redundancy reduction takes precedence over capturing every DFT minimum.

### 3.3 Principal Component Analysis

Although the descriptor-based filtering approach showed promising results in distinguishing unique DFT minima, its main limitation lies in the lack of flexibility to customize the balance between accuracy and computational cost, i.e., various pruning methods were utilized, but further downstream selection is based on two descriptors selected by chemical intuition. To address this limitation, a new method was developed, leveraging all descriptors calculated during the low-level CREST exploration. Since conformers often converge to the same DFT minimum after optimization, it can be hypothesized that such conformers share underlying similarities detectable from the CREST-derived descriptors. Energy alone did not prove sufficient as a distinguishing feature; therefore, we employed a more advanced data-driven method to identify potential similarities among conformers.

This data-driven approach combined dimensionality reduction techniques with clustering methods to identify patterns among the CREST-derived conformers. Dimensionality reduction techniques, such as PCA, are commonly employed on molecular descriptors to facilitate the exploration of chemical space.^9,59,62^ It was hypothesized that the variation in physicochemical properties captured by the descriptors contains information about the behavior of the refined DFT ensemble. As a result, the PCA space was expected to provide a more intuitive way to cluster conformers that are refined to similar DFT geometries. PCA was performed on the complete set of descriptors derived from the CREST-based structures, which included the full set of descriptors (see Data Availability section for the descriptor data set) and the RMSD values of the conformers. Figure 5 presents the chemical space derived from xTB-calculated features following PCA dimensionality reduction, with the coloring indicating the corresponding DFT minima of the conformers. In an ideal scenario, the PCA-reduced space would effectively capture the underlying DFT-defined energy minima, resulting in conformers with identical colors forming distinct clusters. However, the results reveal that this is not the case: the red-colored conformers fail to cluster cohesively, and similarly, the blue-colored conformers in Figure 5b are dispersed across two separate regions. These findings indicate that clustering within the PCA space does not yield an optimal selection of conformers. Furthermore, this observation underscores that the variability in the xTB-derived descriptors does not align well with the stability of conformers as determined by DFT calculations.

**Figure 5:**
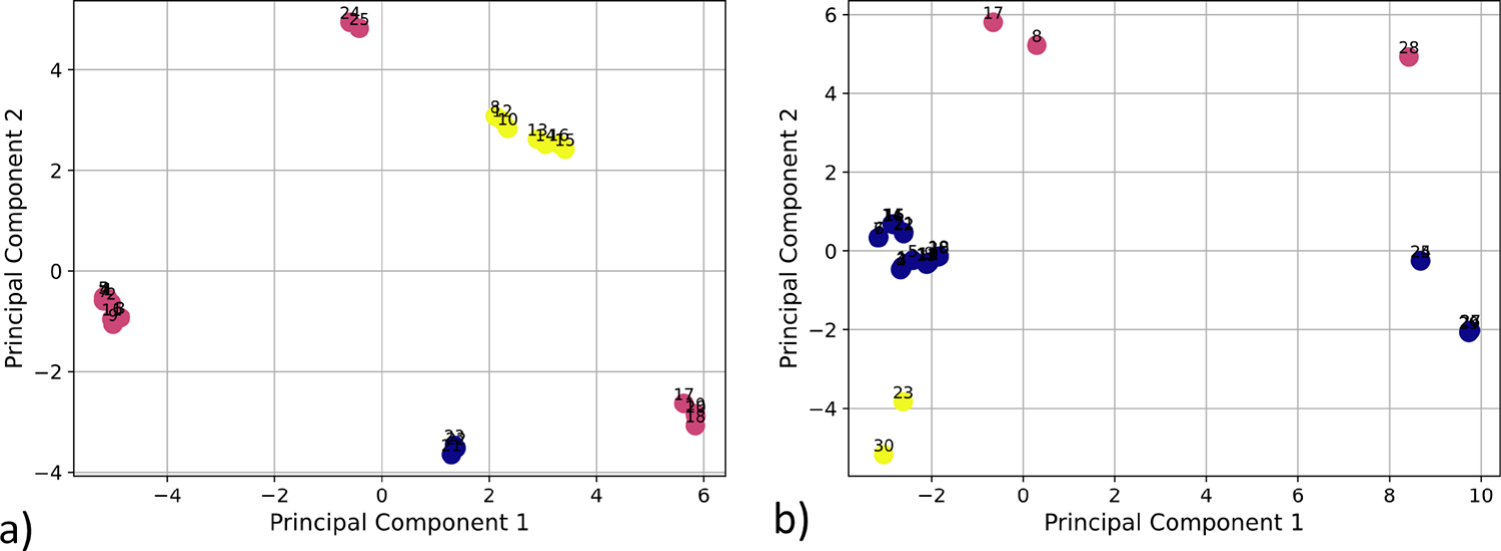
PCA plots of 2 ensembles: ensemble L3 (a) and L20 (b), conformers that converge into the same DFT minimum are marked with the same color.

### 3.4 Clustering

The PCA analysis did not provide a feasible alternative to the previously discussed descriptor-based methods, indicating that incorporating chemical heuristics, such as filtering based on chemically intuitive descriptors, remains preferable. While the descriptor-based methods demonstrated efficiency, they suffer from a lack of flexibility in tuning the size of the ensemble for specific requirements. Additionally, these methods rely on molecular descriptors derived from CREST ensembles, which consequently adds an additional step to the workflow.

Building on the limitations of descriptor-based methods and PCA-based analysis, we explored an alternative filtering approach using unsupervised clustering techniques. Unlike previous methods that relied on a set of descriptors, this new approach focuses solely on the relative energy and RMSD values of the CREST-based conformers. By doing so, it captures both geometric and energetic features without the need for additional descriptor calculations, based on the assumption that conformers with similar geometries and energy values are likely to converge to the same DFT local minimum. An initial comparison of three clustering algorithms, K-means, K-medoids, and DBSCAN, revealed that DBSCAN is best suited for our data set and objectives. Unlike K-means and K-medoids, which allocate all conformers to a cluster and thereby risk excluding key conformers, DBSCAN is designed to manage data with higher noise levels. Conformers are grouped only if they are sufficiently close in RMSD and energy, minimizing the likelihood of overlooking essential conformers in the ensemble. In particular, the cluster size parameter (ϵ) in DBSCAN provides a powerful mechanism to control the definition of “closeness”, enabling the method to be fine-tuned for various objectives. This flexibility allows DBSCAN to strike a balance between precision and computational efficiency in conformer selection.

The results of the DBSCAN clustering (Figure 6a) show that the choice of the ϵ parameter and therefore the size of the clusters significantly influences the performance of the clustering model. The clustering results can be categorized into three parts based on the value of false negatives. In the initial range of ϵ, all DFT minima are successfully captured. As the cluster size increases, the number of redundant conformers eliminated also increases proportionally. At ϵ = 0.19, 369 redundant conformers are filtered out, slightly surpassing the previously reported RMSD pruning method (364) and significantly exceeding the energy pruning method (240). However, as the cluster size is further increased, at ϵ = 0.20, one DFT minimum remains uncaptured, specifically the global DFT minimum of ensemble L8 (see Figure 6b). Increasing the parameter further to ϵ = 0.23 results in an additional missed DFT minimum, which corresponds to the highest energy minimum of ensemble 14. Despite its simplicity, this method outperformed all previously tested approaches while allowing for more precise performance tuning through the parameter ϵ. When capturing all DFT minima is critical, a lower ϵ value can be selected, with the filtering objective gradually shifting from accuracy toward cost-efficiency as ϵ increases.

**Figure 6:**
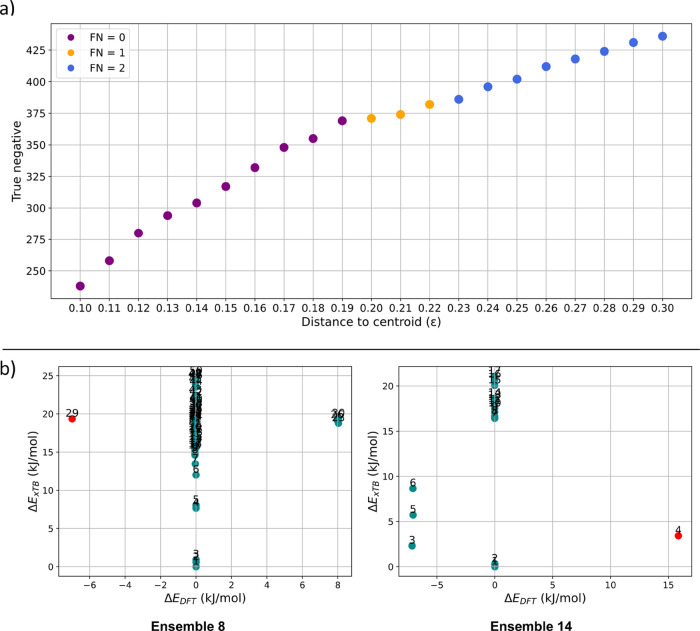
Results on DBSCAN clustering. (a) Results of DBSCAN clustering on the data set of 24 ensembles are presented. The *x*-axis represents the distance to centroid (ϵ) parameter, while the *y*-axis displays the true negative values. Data points are colored according to their false negative values: purple points indicate FN = 0, orange points represent FN = 1, and blue points correspond to FN = 2. (b) CREST-DFT relative energy plots are provided for three ensembles, where DFT minima are potentially missed, with conformers associated with a missed DFT minimum marked in red.

### 3.5 Validation

Although the clustering approach demonstrated promising results on the data set, its applicability to a set of systems with higher conformational flexibility remains uncertain. To assess its generalizability, we validated the method using a data set featuring methyl 2-acetamidoacrylate as the substrate. Switching from the precatalyst to the actual substrate increases the ligand’s flexibility, resulting in a more complex and diverse conformational space.^11^ This increased complexity provides a robust test for evaluating the transferability of our filtering approach and examining the sensitivity of the ϵ parameter across different structural types.

The 11 input structures, reflecting various ligand configurations, yielded 44 CREST ensembles, resulting in a total of 1271 conformers. Following DFT geometry optimization, the refined ensembles contained 154 conformers, indicating that 1117 of the CREST conformers were redundant. Given that DBSCAN clustering within the range of ϵ = 0.10 to 0.19 successfully captured all DFT conformers from the original data set, this algorithm was applied again with the same parameters. The outcome of this clustering approach is illustrated in Figure 7, which plots the ϵ parameter against the number of successfully eliminated redundant conformers. The color of the data points denotes the number of missed DFT minima. These results indicate that even in the best-case scenario with an epsilon value of 0.12, at least one DFT minimum remains uncaptured. However, given the larger number of DFT minima, this shortfall is proportionally less significant. When comparing the number of redundant conformers eliminated across both data sets using the same DBSCAN filtering approach (ϵ = 0.19), it becomes evident that although more redundant conformers are eliminated in absolute terms from the acrylate substrate data set than from the original NBD substrate data set, the relative reduction is lower. Specifically, 462 out of 1117 redundant conformers (41%) were removed from the acrylate substrate data set, compared to 369 out of redundant 644 conformers (57%) in the NBD data set. These findings suggest that although the acrylate substrate data set exhibits more variations in the space of RMSD versus energy at the xTB level of theory, resulting in less straightforward clusters, our approach remains effective. A majority of DFT minima are captured via this simple clustering approach solely based on RMSD and relative energy as metrics.

**Figure 7:**
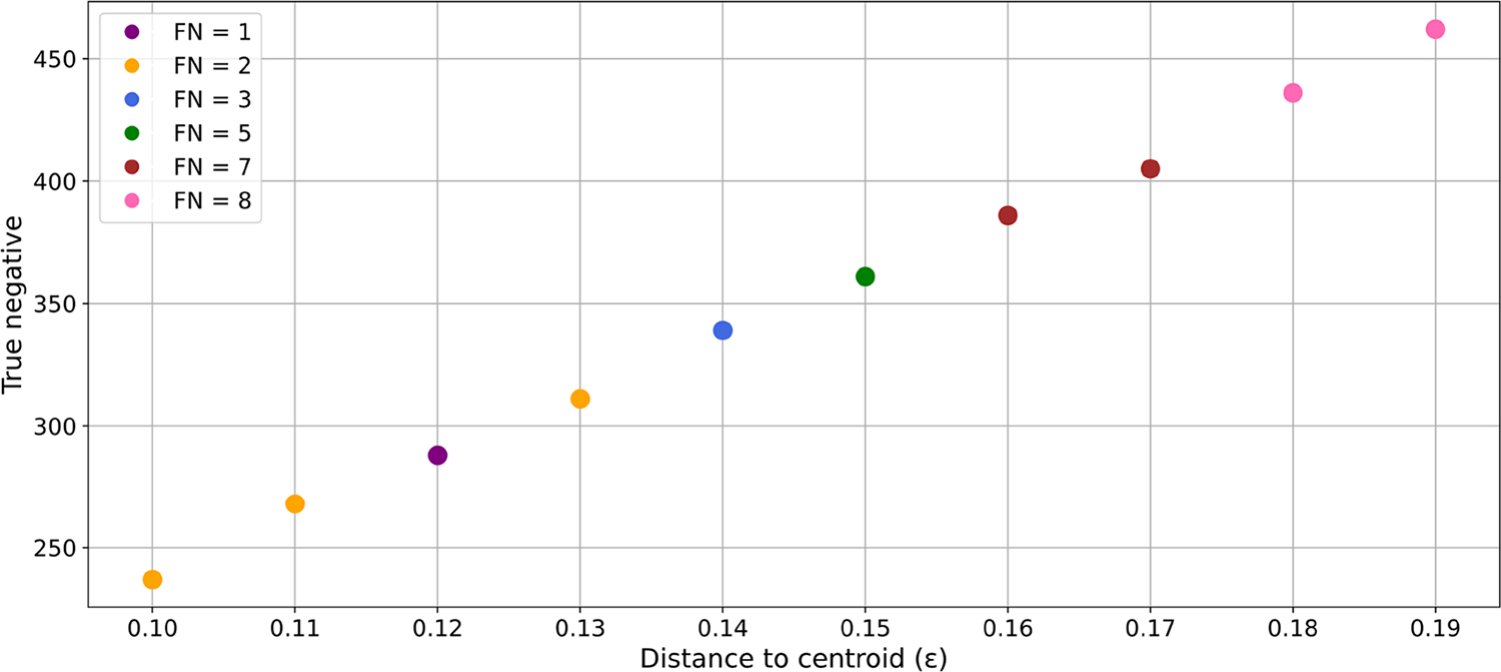
Results of DBSCAN clustering on the validation data set of 44 ensembles are presented. The *x*-axis represents the distance to centroid (ϵ) parameter, while the *y*-axis displays the true negative values. Data points are colored according to their false negative values: purple indicates FN = 1, orange represents FN = 2, blue corresponds to FN = 3, green denotes FN = 5, brown indicates FN = 7, and pink represents FN = 8.

## 4 Conclusions

Computer-readable representations of catalysts enable ML-based screening of widely utilized TM catalysts. The inclusion of conformational flexibility within these representations remains largely dependent on human decisions and assumptions for the filtering of ‘relevant’ conformers. Additionally, less accurate semiempirical or force-field based approaches are preferred over DFT-based methods for the generation of these conformer ensembles due to lower computational cost. This study explored data-driven approaches to correlate conformer ensembles of a lower level of theory to their DFT optimized counterparts, enabling automated filtering of conformers. A data set of 24 precatalyst structures based on our previous research was established for which conformer searching via CREST and subsequent DFT optimization of every resulting conformer was performed. The investigation was performed in three parts. First, a combination of pruning and conformer selection based on geometric ligand descriptors was tested. Afterward, a fully data-driven approach via PCA was tested for the mapping of the CREST-based conformers to their DFT optimized equivalents. Finally, RMSD- and energy-based clustering using DBSCAN was tested and then evaluated on a second data set containing the same ligands, but the precatalyst structure was changed for one containing an acrylate substrate, inducing higher ligand flexibility.

Our research showed that the CREST-generated conformers, when compared to the DFT ensemble, do not reflect the flexibility of the structure. It proved difficult to identify the lowest energy conformer within a DFT optimized conformer ensemble directly based on the energy as calculated in CREST with the GFN2-xTB method. Additionally, CREST produced significantly more conformers compared to the DFT-based ensemble, thus overestimating the flexibility of ligands. Pruning methods demonstrated that pruning based on geometry, rather than energy, resulted in a more accurate mapping to the DFT-based ensemble. This highlighted issues with CREST’s energy calculations and the limitations of energy-based filtering. A fully geometry-based filtering method, using RMSD pruning and selection based on geometric descriptors, outperformed energy-based approaches. However, limitations remained such as limited tunability of this method and one of the DFT minima remaining uncaptured. Unfortunately, a second filtering approach using PCA on descriptors from the CREST ensembles failed to differentiate conformers based on their DFT energy. Remarkably, the simplest algorithm, clustering based on RMSD and energy values, performed exceptionally well. DBSCAN clustering with these features showed the best filtering, with the lowest false negative rate and the highest elimination of redundant conformers. This method can be fine-tuned using the cluster centroid distance parameter, balancing accuracy and computational cost for different applications. It also does not require the calculation of molecular descriptors for the CREST ensemble. When tested on a validation data set containing an acrylate substrate with increased ligand flexibility compared to that of a precatalyst structure, the method remained effective, suggesting its general applicability across various catalyst structures employing bisphosphine ligands.

Overall, our findings bear significance for the dynamic representations involving conformational flexibility of catalyst structures in high-throughput virtual screening workflows. A shortcoming of this approach is that the relationship between the distance to centroid parameter and the resulting accuracy-cost trade-off is highly dependent on the chemical structures themselves, making it challenging to tune. Additionally, when a very high accuracy is required, e.g., for the approximation of enantioselectivity, filtering based on constant energy refinement and reweighting conformers would be more advisable. Developments in conformer filtering approaches as researched in this study go hand-in-hand with developments in the field of conformer searching methods,^22,63–65^ ML-based energy calculations,^66^ and more efficient exchange-correlation functionals^67,68^ where constant improvements are being made in the chemical space of transition-metal complexes. Nevertheless, a DBSCAN-based clustering approach utilizing the xTB-based energy and RMSD remains the most simple and computationally feasible option for now. This approach is being utilized in our current and future research on dynamic representations of homogeneous catalysts for ML-based virtual screening.

## Data Availability

The Python package for the featurization of catalyst structures, OBeLiX, is available through the GitHub organization page of the ISE group at TU Delft: EPiCs-group OBeLiX (https://github.com/EPiCs-group/obelix), with the specific version to calculate descriptors for individual conformers from a CREST ensemble contained on a separate branch (https://github.com/EPiCs-group/obelix/tree/confomer_searching_dev_final). All data sets used in this study are provided with an extensive README via 4TU. ResearchData at https://doi.org/10.4121/45bb4e4b-272b-41ce-a090-2b6e4b1708fd. The following resources are included: A list and visualization of ligands (‘ligand_description.docx’). An Excel file categorizing and describing all descriptors (‘descriptors_description.xlsx’). Script for conformer filtering and creating figures used for analysis (‘data_analysis.py’). Pickled ConformerEnsemble objects created with the Morfeus package containing conformers, xTB energies and RMSD values (‘conformer_ensemble_files.zip’). Input and output of conformer searching with CREST (‘CREST_structures.zip’). CSV files with descriptors for each conformer calculated at the xTB level of theory (‘descriptors.zip’). Input and output of DFT optimized files with Gaussian 16 (‘DFT_structures.zip’). MDL Molfiles to extract the connectivity matrix per metal–ligand complex for conformer searching and analysis (‘mol_files.zip’). Files with energy values and tracking which conformers are pruned in a conformer ensemble (‘pruning_files.zip’). Data from the case study on a validation set from our previous research (‘validation.zip’). Figures for PCA-, clustering- and energy-based conformer selection approaches (‘visualization.zip’).
